# Properties of Yoghurt Fortified in Lactoferrin with Effect of Storage Time

**DOI:** 10.3390/ani13101610

**Published:** 2023-05-11

**Authors:** Anna Jańczuk, Aneta Brodziak, Jolanta Król, Tomasz Czernecki

**Affiliations:** 1 Department of Quality Assessment and Processing of Animal Products, Faculty of Animal Sciences and Bioeconomy, University of Life Sciences in Lublin, Akademicka 13, 20-950 Lublin, Poland; jolanta.krol@up.lublin.pl; 2 Department of Biotechnology, Microbiology and Human Nutrition, Dietitian Service, Faculty of Food Science and Biotechnology, University of Life Sciences in Lublin, Skromna 8, 20-704 Lublin, Poland; tomasz.czernecki@up.lublin.pl

**Keywords:** milk proteins, fermented products, quality, physical-chemical properties, organoleptic characteristics, texture, water activity, innovative product

## Abstract

**Simple Summary:**

Lactoferrin is a whey protein with a high health-promoting potential, also in the context of civilization diseases such as obesity and type 2 diabetes. It is perfectly suitable as an addition to fermented milk drinks—yoghurts, which are easily acceptable in the diet. As a natural milk protein, it is perfect for increasing the health-promoting value of yoghurts. The results obtained from the physicochemical, microbiological, and organoleptic assessment of yoghurts indicated that the use of yoghurt fortification with lactoferrin in the amount of 80 mg/100 g in most cases did not statistically significantly affect the analysed parameters. Yoghurt with the addition of lactoferrin was fully acceptable by the testers.

**Abstract:**

The stability of fortified yoghurts during refrigerated storage is important for industry and the consumer. The aim of the study was to evaluate the nutritional value, microbiological quality, organoleptic properties, and structure of natural yoghurts made with the addition of lactoferrin during refrigerated storage. In this study, we produced natural yoghurts fortified in lactoferrin, using YC-X11 yoghurt starter culture based on *Lactobacillus delbrueckii* subsp. *bulgaricus* and *Streptococcus thermophilus*. Physicochemical (acidity, nutritional value and structure) as well as microbiological and organoleptic changes occurring during 28-days refrigerated storage were determined. Storage research made it possible to determine the direction of changes taking place in the products. The analysed parameters did not differ statistically significantly between the control yoghurts and those with the addition of lactoferrin. Textural and rheological studies also shown that the addition of lactoferrin did not significantly change the structure of the yoghurt. The yoghurts were characterized by high sanitary and hygienic quality during the whole refrigerated storage. Lactoferrin has a positive effect on the durability of the product.

## 1. Introduction

According to the recommendations of the Food and Agriculture Organization (FAO) in cooperation with the World Health Organization (WHO), European citizens should consume daily milk and dairy products (kefir, sour milk, yoghurt, cheese) due to its high nutritional value and beneficial health effects on the human body [1]. Milk proteins are considered the richest source of nutritional and functional ingredients in the diet. Nutritionally, they are a building material and a source of essential amino acids for normal growth and development of the body. The functional properties of proteins, including water binding capacity, gelling, emulsifying or foaming properties, affect the physicochemical and organoleptic properties of the food produced [2,3]. Milk proteins also have important biological functions. One of the biologically active milk proteins is lactoferrin (LF), classified as a whey protein. Its presence in the highest amount was found in milk, but also in many organs (liver, kidneys, pancreas, lungs, gallbladder, prostate, seminal vesicles, intestines), serous membranes and their secretions [4]. The lactoferrin content in cow milk ranges from 50 to 120 mg/L. Human milk contains the highest content of LF, but it should be noted that it has a significantly lower biological activity than milk-derived LF from other mammalian species [4,5,6]. To date, a number of its biological properties have been demonstrated, including binding of iron, calcium, copper, aluminium or manganese ions, bactericidal, bacteriostatic, antifungal, antiviral, antiparasitic, antioxidant and anticancer activity, as well as being an organ non-specific immunity component, cell growth regulator, and precursor of bioactive peptides [4,5,6,7]. Studies in recent years have shown that LF exerts a protective effect in the course of obesity and improves insulin sensitivity, making it an effective component of dietary supplements for people struggling with excessive body weight, including those with a predisposition to obesity [8,9]. Its use in the prevention of cardiovascular diseases [10,11], treatment of iron deficiency anaemia or iron deficiency [12], oxidative stress reduction [13] or modulation of intestinal microflora composition [14] has also been indicated. Research by the New Zealand pharmaceutical manufacturer Quantec demonstrated that a protein complex containing LF and lactoperoxidase could protect human cells against COVID-19. The patented IDP defence protein showed anti-inflammatory, antioxidant and antibacterial properties [15].

Due to these unique properties of LF, techniques for its separation and purification from cow milk or whey have been developed for years. This is facilitated by advances in milk protein separation techniques [16,17]. Despite the high cost of its isolation, LF is in high demand on the market. LF preparations are used to enrich the nutritional or health value of food, mainly fermented dairy products. In 1997, yoghurts fortified with bovine LF appeared on the Japanese market. Importantly, on 22 November 2012, the European Commission authorized the marketing in the EU of bovine LF (bLf) as a novel food ingredient [18]. Moreover, in accordance with the European Food Safety Authority (EFSA), LF derived from cow milk is safe under appropriate conditions of use, standards and levels. Since then, in addition to yoghurts and infant formulas originally available on the Japanese market, many new products fortified with LF have appeared on the European shelves, including juices, water and chewing gum. Products containing LF can be used virtually without restrictions, although undesirable side effects of consuming this protein have been observed, but after taking significant doses exceeding 2000 mg/kg body weight. For this reason, EU regulates [19] the maximum level of bLF applied in food. The maximum addition of bLF in milk-based beverages is 200 mg per 100 g, and fermented milk-based drinks (including yoghurt drinks) can contain up to 50 mg of LF in 100 g, yoghurt-based products—up to 80 mg in 100 g, cheese-based products—up to 2000 mg per 100 g, and ice cream—up to 130 mg per 100 g. In turn, food for special medical purposes can provide up to 3 g of LF per day, depending on the individual patient’s needs. It should also be noted that due to its antimicrobial properties, it can serve as a natural preservative for dairy products, thereby reducing the use of additives, especially preservatives. This is an additional premise for enriching dairy products with this compound. Importantly, experimental studies have demonstrated that LF does not exhibit antimicrobial activity against probiotic bacteria, nor does it affect the viability of lactobacilli, as these bacteria do not require iron for growth. In the case of bacteria of the genus *Bifidobacterium*, lactoferrin stimulating effect on the bacterial cell population is based on supplying them with iron ions.

Currently, there are cosmetics and pet food available in the world that contain this protein, but primarily it is supplemented to pharmaceuticals, including dietary supplements. It should be emphasized that dietary supplements containing LF are available on the Polish market. However, it is not currently used as a food ingredient, despite being approved for marketing.

The available knowledge enables the rapid development of the food market. Increasingly, food manufacturers offer products that not only meet the basic nutrient requirements, but also have programmed health-promoting properties. Bioactive ingredients can be incorporated into the product, taking into account population health risks, as well as individual characteristics of consumers. The aforementioned documented properties of LF enable its conscious use in food products dedicated to consumers with health problems resulting from genetic predispositions and/or environmental influences. This provided the rationale for conducting research and proposing a food product fortified with LF. Only a few papers have been published so far on the possibility of using LF in dairy products, including one on cheese [20] and three on yoghurts, one of which [21] is focused on the effect of LF of different iron saturation on yoghurt acidity and growth of lactic acid bacteria during fermentation and storage; the second [22] concerns the effect of LF on pathogenic bacteria and amino acids in yoghurt fortified with buffalo, bovine, mix colostrum and LF colostrum; the third [23] is devoted to the effect of donkey milk LF and lysozyme on selected yoghurt properties. The aim of the research was, therefore, to produce the yoghurt based on cow milk fortified with an acceptable amount of lactoferrin in order to comprehensively determination its physicochemical, microbiological and organoleptic characteristics with regard to storage time. The authors intend to propose a specific product to the market and ascertain whether LF will integrate with yoghurt and whether its addition at the maximum permissible level will change the properties, and acceptability during its shelf life, usually defined in dairy practice as 28 days. Yoghurts are widely known and eagerly consumed by consumers, which makes them a good carrier of beneficial bacteria or bioactive compounds.

## 2. Materials and Methods

### 2.1. Research Material

The pasteurized bulk cow milk for yoghurt production was obtained from the dairy located in Lublin Voivodeship, Poland. Raw material was normalized (3.2% fat content) and pasteurized (very high temperature (VHT) pasteurization: 85 °C for 20–25 s). It was transported to the laboratory under refrigeration, and immediately used for yoghurt production. Milk was collected from the dairy three times.

### 2.2. Yoghurt Production

**Figure 1:**
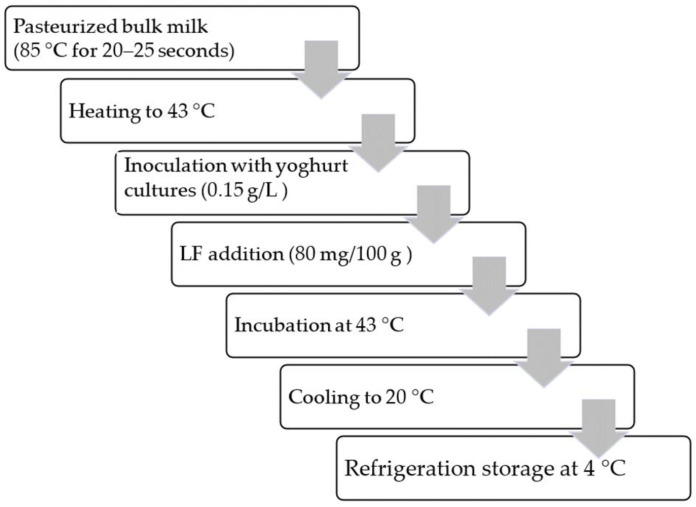
The yoghurt manufacturing method.

The milk was heated to 43 °C and inoculated with 0.15 g/L of FD-DVS YC-X11 Yo-Flex, containing *Streptococcus thermophilus* and *Lactobacillus delbrueckii* subsp. *bulgaricus*—thermophilic yoghurt cultures from Chr. Hansen (Graasten, Denmark) [24], Figure 1. Certified lactoferrin (Biolive Innovation, Lublin, Poland) in the amount of 80 mg/100 g [19] was used to enrich the yoghurt. Unfortified yoghurt served as the control group. The inoculated milk was incubated in polypropylene (PP) plastic 100 mL containers at 43 °C (according to the manufacturer’s instructions) until pH = 4.6 was attained (approx. 5 h). The products were then immediately cooled to 20 °C to discontinue the incubation. The yoghurts were stored at 4 °C until the next day (approx. 16 h) for analysis. The analyses were performed every 7 days, i.e., on day 0, 7, 14, 21 and 28, during refrigerated storage at 4–6 °C. A total of 210 yoghurt samples were prepared, i.e., three times per 70 samples each when milk was collected. In total there were 105 plain, natural yoghurts and 105 yoghurts with LF addition. In each yoghurt type, 21 samples were analysed at each day of storage, of which 3 for basic chemical composition and LF content, 3—microbiological evaluation, 3—texture and colour parameters, 3—water activity and water-holding capacity, 3—syneresis and acidity, and 6—organoleptic quality. In each day of storage new, unopened samples were analysed.

### 2.3. Yoghurt Analysis

#### 2.3.1. Basic Composition and Acidity

The yoghurts were analysed for the content of protein (Kjeldahl method according to PN-EN ISO 8968-1:2014 [25] using the mineralizer Tecator Digestor Auto 20 (FOSS Analytics, Hillerød, Denmark) and the automatic analyser KjelROC (OPSIS LiquidLINE, Furulund, Sweden); fat (the weight method using the Soxtec Avanti^®^ extraction unit (Tecator, FOSS Analytics, Hillerød, Denmark) and the PS 750/X analytical scale (Radwag, Radom, Poland)); and dry matter (oven-drying at 102 °C using the laboratory dryer (Memmert, Schwabach, Germany) [26]. The measurements were taken in triplicate. The energy was calculated on the basis of the individual basic components.

#### 2.3.2. Lactoferrin

In order to evaluate the content of lactoferrin, RP-HPLC method was used. All samples were prepared according to Brodziak et al. [27] with a modification (5 mL of yoghurt instead of milk). Protein analysis was performed on liquid chromatograph ProStar 210 model and UV-VIS ProStar 325 detector (Varian, Palo Alto, CA, USA). The measurements were carried out using the water/acetonitrile mobile phase at gradient elution and column Nucleosil 300-5 C18 (Varian, Palo Alto, CA, USA) of 250 mm length and 4.6 mm diameter. The mobile phase was solvent A (90% water, 10% acetonitryle) and solvent B (90% acetonitryle, 10% water), purchased from Sigma-Aldrich (St. Louis, MO, USA). The analysis time for a single sample was 15 min at 205 nm wavelength with column temperature of 37 °C. The analysis of reference substance—commercially available lactoferrin (purity—90%, Sigma-Aldrich (St. Louis, MO, USA)), was conducted under the same conditions. On the grounds of the obtained chromatograms, the qualitative and quantitative identification of LF was performed.

#### 2.3.3. Acidity

The active acidity (pH value) was measured before, during and after fermentation using a CP-401 pH-meter (Elmetron, Zabrze, Poland). The measurements were taken in triplicate. The potential acidity was determined by the titration method [28] and expressed as lactic acid content in %.

#### 2.3.4. Microbiological Evaluation

Prior to the microbiological evaluation, 10 g of each yoghurt sample was placed in sterile bottles containing 90 mL of Ringer’s solution [29,30]. Nextly, it was shaken for 5 min and allowed to sediment for 15 min. Then, a series of ten-fold dilutions of the samples were prepared in Ringer’s solution and spread onto previously prepared Petri dishes with an appropriate microbiological medium. The following was determined in each material: total number of mesophilic aerobic bacteria—on fortified agar medium for 48 h at 30 °C (BTL Ltd., Warsaw, Poland) according to PN-ISO 7889:2007 [31]; total number of fungi—on Sabouraud medium for 5–7 days at 30 °C (BTL Ltd., Warsaw, Poland) [32]; total number of lactic acid bacteria of the genus *Lactobacillus* sp.—on MRS medium for 3–5 days at 30 °C (BTL Ltd., Warsaw, Poland) [33]; total number of *Clostridium perfringens*—sulfate reducing bacteria (IV) growing in anaerobic conditions on Iron sulfide agar (TSC) for 48 h at 37 °C (Biomerieux Poland Ltd., Warsaw, Poland) [34]; total number of coliform bacteria on Endo les medium for 24 h at 37 °C (BTL Ltd., Warsaw, Poland) [35]; total number of fecal *Escherichia coli*—on mFC medium, for 18–24 h at 44 °C (BTL Ltd., Warsaw, Poland) [35]; *Campylobacter* bacilli [36]; presence of *Salmonella* bacilli—on SS medium (*Salmonella-Schigella* and XLD) after prior sample multiplication in buffered peptone water and Rappaport-Vassiliadis medium (BTL Ltd., Warsaw, Poland) for 24 h at 37 °C [37]. Final identification was performed using the available API tests (BioMerieux Poland Ltd., Warsaw, Poland) and polyvalent sera (Biomed S.A., San Antonio, TX, USA). Each previously prepared sample was plated in triplicate. After incubation, the colonies were counted using an automatic Scan 300 counter (Interscience Laboratories, Saint-Nom-la-Bretèche, France) and the number of individual morphological types was counted as the number of colony forming units in 1 g of the yoghurt (cfu/g).

#### 2.3.5. Texture

The texture parameters of the yoghurt curds (firmness, consistency and cohesive strength) were measured using a BDO-FB0.5TS universal testing machine (Zwick GmbH and Co., Ulm, Germany). The yoghurt curds were prepared in a dedicated beaker (50 mm in diameter and 150 mm high). For each yoghurt curd, two yoghurt samples were prepared and tested after approximately 20 h of storage at 4–6 °C. A beaker with the sample was centrally placed under the plunger of the apparatus with a cylindrical die 45 mm in diameter and 5 mm in height, and then compressed to a depth of 25 mm at a speed of 1 mm/s. On the basis of the force-time curves obtained, the following texture characteristics were determined for the curds: firmness—the maximum positive force (N), consistency—the positive area of the curve up to the maximum point (mJ) and cohesive strength—the maximum negative force (N). The measurements were taken in duplicate. The results of the measurements were processed using TestXpert® II software (2011, Zwick GmbH and Co, Ulm, Germany). Rheological measurements—dynamic viscosity (mPa·s) were conducted using the HAAKE Viscotester iQ rheometer (Thermo Fisher Scientific, Walthman, MA, USA) equipped with an cylindrical rotor. The measurements were taken in duplicate, at 10 °C.

#### 2.3.6. Water Activity

The water activity (w_a_) in the yoghurts was measured using a HygroLab C1 water activity meter (Rotronic, Bassersdorf, Switzerland). Measurements were conducted using the AWQ mode and stabilization for 15 min after the yoghurts had reached room temperature. The determinations were made in triplicate.

#### 2.3.7. Water-Holding Capacity and Syneresis

The water-holding capacity—WHC was determined [38]. 10 g of yoghurt were weighed into a test tube and then centrifuged in a laboratory centrifuge (UNIVERSAL 320; Hettich, Tuttlingen, Germany) at 5 °C for 10 min at 1250× *g*. After the indicated time, the precipitated whey was weighed. The tests were carried out in triplicate. WHC was calculated based on the formula:

WHC (%) = (10 − W)/10 × 100%,

where: W—mass of the separated whey (g).

Spontaneous whey syneresis—SWS was also measured according to Narayana and Gupta [39] and Cais–Sokolińska and Walkowiak-Tomczak [40]. A plastic container with 100 mL of yoghurt was stored at 4–6 °C and immediately after removal from the refrigerator, it was tilted at an angle of 45° to collect the surface whey using the syringe. It was performed within 10 s to avoid a whey forced leakage from the gel. SWS was expressed directly as the volume of whey expelled by 100 mL of yoghurt, in %.

#### 2.3.8. Colour Measured Instrumentally

The colour of the yoghurt was measured by a Minolta CR-310 Chroma Meter (Minolta Camera Co. Ltd., Osaka, Japan), using D65 as the standard light source. The measurements were carried out directly in the plastic containers in which the yoghurt was stored. The reflectance of the yoghurt surface was measured using a measuring head (50 mm aperture diameter; geometry 0°). The CIE colour parameters were the following: L* (lightness), a* (redness/greenness) and b* (yellowness/blueness). The colour tests were performed in four replications [41].

#### 2.3.9. Organoleptic Assessment

An organoleptic assessment of the yoghurts was performed by a suitably prepared 10-person panel. The panel consisted of 7 women and 3 men in the age 25–45 years, who specialize in this type of assessment. People had been trained in the assessment methodology according to ISO 4121 [42]. Prior to the evaluation, the samples were coded and left to stand for 1 h at room temperature to reach a suitable temperature for eating, and then they were presented to the testers together with a questionnaire. Each person occupied a separate place in the room to prevent a mutual communication. A five-point scale was used to evaluate the following: colour, consistency, flavour, aroma and general acceptance of the products, in which 1 designated disqualifying quality (unsuitable) and 5 indicated very good quality (completely acceptable, characteristic for the product) [43].

### 2.4. Statistical Analysis

A statistical analysis of the results was performed using two-way analyses of variance—ANOVA (StatSoft Inc. Statistica ver. 13.1; Dell, Round Rock, TX, USA). The significance of the differences between the means for the groups was determined by Kruskal-Wallis’s test at a level of *p* (alpha) = 0.05 and 0.01. The results are presented as the means ± standard deviation (SD).

## 3. Results

### 3.1. Nutritional Value

The nutritional value of a food product, which is of increasing interest to consumers, is determined by the presence of essential nutrients for the functioning of the human body, whose quantity or bioavailability may be reduced by complex technological processes and other factors. Table 1 presents the results pertaining to the basic nutritional value of the yoghurts, i.e., the content of crude protein, fat, and dry matter.

The use of the maximum permissible level of LF did not significantly (*p* > 0.05) influence the parameters analysed. Yoghurt with the addition of LF contained 12.36% dry matter, including 3.22% crude protein and 3.25% fat. These levels were comparable to those in the control yoghurt, without the addition of this protein. The 28-day storage period in refrigeration conditions did not significantly (*p* > 0.05) influence the content of fat. In the case of crude protein and dry matter, the differences in this type of yoghurt depending on the day of storage were statistically significant (*p* ≤ 0.05). In general, the content of all nutrients decreased with the passage of storage time, possibly due to the type of starter cultures used (their activity) and enzymatic processes [44]. A study using donkey milk lactoferrin and lysozyme in yoghurt production [23] also showed a decrease (of 0.1%) in the content of fat and dry matter during 30-day storage of the control yoghurt and three experimental yoghurts (yoghurt treated with lactoferrin, yoghurt treated with lysozyme, and yoghurt treated with natamycin), although the differences were not statistically significant (*p* > 0.05). In yoghurt treated with LF, the crude protein content increased during storage (from 4.05% to 4.36%, *p* > 0.05). It should be noted, however, that for the control yoghurt, yoghurt treated with lysozyme, and yoghurt treated with natamycin, the direction of changes was the same as in the present study, i.e., the amount of protein decreased during storage (by 0.58, 0.15 and 0.25 p.p., respectively). However, the authors did not explain the cause of these changes. Another study, analysing the addition of whey protein concentrate to yoghurt [45], found comparable levels of protein and fat on the first (day 0) and last (day 28) days of refrigerated storage. However, the content of non-fat dry matter and total dry matter decreased.

The energy value of the yoghurts was comparable, irrespective of the type and storage time, at 62–64 kJ/100 g of product. This value is comparable to that of commercially available natural yoghurt.

### 3.2. Lactoferrin

Since the yoghurt was fortified, i.e., the content of a component naturally occurring in milk was increased by adding 80 mg of LF, in compliance with the regulation [19], the lactoferrin level was assessed as well. It was 47.69 mg/100 g in the control yoghurt and 131.15 mg/100 g in the fortified yoghurt (Table 2). LF is naturally present in raw cow milk in the amount of 0.1–0.3 mg/mL, but during heat processing this level decreases. In other studies [46] it was noticed that the type of heat treatment significantly influenced the content of non-denatured whey proteins in drinking milk. The most valuable source of whey proteins, particularly lactoferrin and lysozyme, was the micro-filtered milk. Claeys et al. [47] reported that only heating at 85 °C for 30 min leads to total inactivation of LF. Moreover, according to Darmawan et al. [48], high temperature induced structural changes of apo-lactoferrin and interactions with β-lactoglobulin and α-lactalbumin. Another study confirmed that LF is more resistant to denaturation when saturated with iron. Binding to iron increases the stability of the protein structure [49]. The effect of LF iron saturation on its stability under high temperatures was reported by Liu et al. [50]. Regardless of the iron content, LF is stable in the pH range from 4 to 11 [51].

The storage period was shown to influence the content of this protein. Irrespective of the type of yoghurt, the content of LF decreased statistically significantly (*p* ≤ 0.05), on average by 14%, during 28-day storage (Table 2). An influence of the storage period on LF content was also reported in the studies devoted to the changes in the content of bioactive compounds in drinking milk [52].

### 3.3. Acidity (pH Value and Lactic Acid)

**Figure 2:**
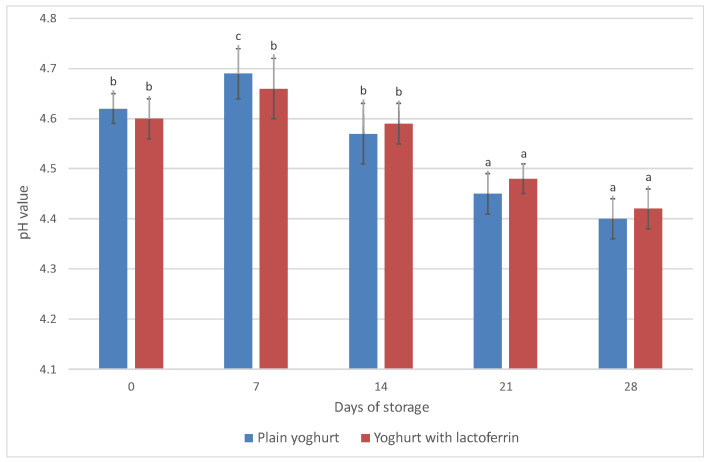
Changes of pH value in the analysed yoghurts during 28 days of storage in the refrigerated conditions. ^a, b, c^—differences between the pH value within a yoghurt type, significant at *p* ≤ 0.05.

**Figure 3:**
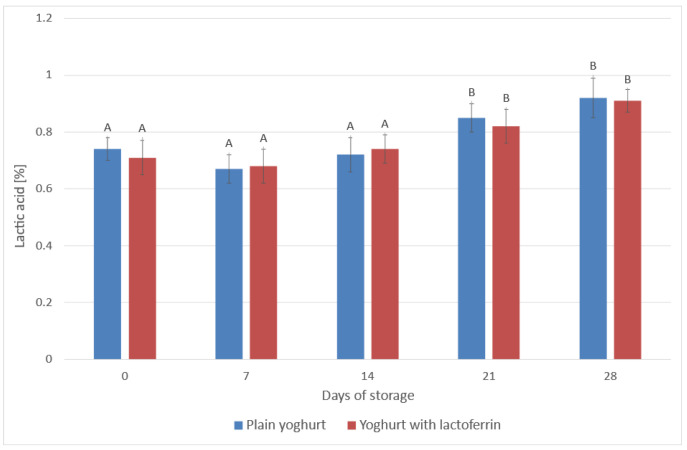
Changes of lactic acid content (%) in the analysed yoghurts during 28 days of storage in the refrigerated conditions. ^A, B^—differences between the pH value within a yoghurt type, significant at *p* ≤ 0.01.

Incubation of the yoghurt was stopped when the pH of the control yoghurt reached approx. 4.60. The variations in pH value (Figure 2) and % lactic acid (Figure 3) were measured throughout the storage period. In Figure 2, the ‘0’ point represents the pH measurement taken on the first day of storage at 4 °C. At the end of the storage period (‘28’), the pH values were 4.41 in the control yoghurt and 4.10 in the yoghurts supplemented with LF. The pH of the yoghurts generally varied within an acceptable range during storage. As expected, the value of this parameter decreased over storage time, and the decrease was statistically significant (*p* ≤ 0.05). Changes in pH are associated with the type of bacterial culture used and the storage temperature. At temperatures above 4 °C the number of fermenting bacteria increases, which reduces the active acidity value [53]. Franco et al. [21] reported that the decrease the authors observed in pH was directly linked to the concentration of LF, because a higher LF concentration caused higher pH values than in the control yoghurts. Assessment of titratable acidity in the present study revealed an increase in the content of lactic acid of about 0.28 p.p. (Figure 3). On the last day of storage, both yoghurts contained just over 1% lactic acid. This parameter was shown to be statistically significantly influenced by the storage time (*p* ≤ 0.05), but not by the addition of LF (*p* > 0.05). Amadarshanie et al. [54] reported a decrease in pH from 4.92 to 3.58 and a twofold increase in the content of lactic acid (to 1.24%) during 21-day storage of control natural yoghurt based on the same strains used in the present study. This is because the content of lactic acid changes in the reverse direction to the change in pH, due to fermentation of lactose by the lactic acid bacteria used to make the yoghurt, which produce lactic acid. The high metabolic activity of the bacteria used to produce yoghurt decreases immediately after incubation due to cooling, but the enzymatic activity of the bacteria continues during the storage period. For this reason changes in acidity are observed even after the incubation period [55]. Arslaner et al. [56] reported smaller differences, with a reduction in the pH of the control yoghurts amounting to 0.15 (from 4.31 to 4.16, *p* ≤ 0.01) and an increase in the content of lactic acid of 0.08 p.p. (from 1.00% to 1.08%, *p* ≤ 0.01) over three weeks. In another study [40], the change in the acidity of natural yoghurts over a three-week period was not significant. Brodziak et al. [45] added WPC to yoghurts and observed an increase in active acidity (a reduction in pH) accompanied by an increase in the content of lactic acid in the fortified yoghurts compared to the controls. The addition of whey proteins increases the buffering properties of milk, which affects potential acidity—lactic acid content. These changes were confirmed statistically during one month of storage. The same tendencies were obtained by Karam-Allah et al. [22] during 10-day storage of yoghurt enriched with buffalo and cow colostrum and lactoferrin, but also by Akal et al. [23] in yoghurt made from donkey milk with the addition of lysozyme, lactoferrin and natamycin. Karam-Allah et al. [22] reported that stirred yoghurts with colostrum had slightly reduced titratable acidity when fresh and during storage, possibly due to the presence of bioactive components (antimicrobial agents) in colostrum and lactoferrin, which decreases the chances of fermentation. Zakaria et al. [57] reported a reduction in active acidity (pH) and an increase in titratable acidity in yoghurt with added whey protein compared to control yoghurt during 7-day storage, which in the case of pH was also confirmed by Karam-Allah et al. [22]. This may be due to partial inhibition of lactic acid by lactoferrin. In the present study, these changes over time were comparable with the control yoghurt.

### 3.4. Microbiological Evaluation

Consumers reaching for new products with proven health-promoting properties must be guaranteed high microbiological quality, which translates into health safety. Research on determining the level of microbiological contamination informs not only about the quality of the product, but also the hygiene of the process and the quality of the additives. Analysing the total bacteria count, it was found that the fresh control and experimental yoghurts did not contain any contaminants (Table 3). However, the changes occurred during the storage of yoghurts without the addition of LF. Bacteria (in total) were detected on day 14 of the study and their count remained at the same level until the end of the storage period. These differences were statistically confirmed (*p* ≤ 0.01). The study also assessed the total number of bacteria of the genus *Clostridium* sp., *Coli* bacteria (Endo), faecal coliforms (mFC), as well as the presence of *Salmonella bacilli* and *Campylobacter bacilli*. They were absent in both the control and experimental yoghurts (Table 3). The microorganisms also did not appear in the products during refrigerated storage. In addition, no fungal growth was observed in the control and experimental yoghurts at any of the analysed time periods. Yoghurts are a source of lactic acid bacteria. In the yoghurts produced, their total number was at the level of 6.9–7.1 × 10^7^ cfu/mL. No statistically significant differences in the number of these microorganisms were recorded throughout the storage period. It should be noted that the yoghurts met the requirements of Commission Regulation (EC) No. 2073/2005 [58] on microbiological criteria for foodstuffs. One of the factors determining the therapeutic, prophylactic and dietary values of yoghurts is the presence of live cultures of starter bacteria (*Streptococcus thermophilus* and *Lactobacillus delbrueckii* subsp. *bulgaricus*) throughout their declared shelf life [59]. According to the requirements of the FAO/WHO *Codex Alimentarius* standard [60], the number of characteristic microflora of yoghurt must be at least 10^7^ cfu in 1 g of the product during its shelf life, which is taken into account in the Polish standard [26], wherein at least 10^6^ should be living lactic acid bacteria. In the study by Franco et al. [21], bacterial counts were virtually constant throughout the entire period (including 28 days), regardless of the form of lactoferrin applied. The mean value for *L. delbrueckii* spp. *bulgaricus* was 6.37 log cfu/mL, and 8.87 log cfu/mL for *S. thermophilus*. LF has been indicated to be highly stable during food storage, including yoghurts. However, Franco et al. [27], in a more detailed study, taking into account the form of LF (different iron saturation), showed some variations in the concentration of bovine apo-lactoferrin during the storage period. According to the authors, this should also be linked to different pH values of the yoghurts, since yoghurt containing apo-lactoferrin had the highest pH value during storage (apo-lactoferrin yoghurt pH > control yoghurt pH > holo-lactoferrin yoghurt pH). Similar studies were also carried out by Kim et al. [61], who found that bovine holo-lactoferrin stimulated the growth of *Lactobacillus acidophilus*. However, in the present study, the forms of LF were not determined, also due to its lack of availability on the market. In assessing the microbiological quality of donkey milk-based yoghurts, Akal et al. [23] considered only the counts of total aerobic mesophilic bacteria and yeasts/moulds. The total number of aerobic mesophilic bacteria was lower in the lactoferrin-treated fresh yoghurt than in the fresh control yoghurt (1.07 vs. 2.54 log cfu/g). In both cases, no yeasts and moulds were found in the fresh product. There was an increase in the total aerobic mesophilic bacteria count during storage, both in the control and experimental yoghurts. The storage period also affected the development of yeasts and moulds in the control yoghurt, which was not observed for the experimental yoghurt (with lactoferrin and lysozyme). Steijns and Hooijdonk [62] indicated that the antimicrobial effect of LF against yeasts and moulds also relied on iron binding, as in the case of bacteria. It should be noted that lysozyme in this study [23] was found to be generally more effective in its antimicrobial activity than LF.

In general, yoghurts with LF addition, due to their antimicrobial properties, will show higher microbiological stability during storage. However, the use of LF of high microbiological purity should be ensured.

### 3.5. Texture

Texture is a very important determinant of the quality and acceptability of yoghurt. The final texture of fermented dairy drinks is determined by their structural arrangement and protein microstructure network [63]. The texture results can be explained by many factors, such as their composition (content of dry matter including protein and fat), the production method, incubation conditions, cooling, the use of added ingredients, and storage conditions. It should be noted that in the water bath method, once the fermented drink is obtained it is no longer possible to interfere with the contents of the package. For this reason it is usually used to produce natural (plain) yoghurt, without added flavourings.

Assessment of texture in the present study took into account firmness, consistency, cohesive strength, and dynamic viscosity. Strains of *L. delbrueckii* subsp. *bulgaricus* and *S. thermophilus* bacteria were used to produce the yoghurts. In the starter mixture *S. thermophilus* is responsible for releasing the aroma, while *L. delbrueckii* ssp. *bulgaricus* is responsible for acidification, which promotes casein coagulation [64]. Both strains synthesize exopolysaccharides (EPS). EPS are secreted out of the cell in the form of slime or bound to the cell surface [65,66]. Research [67] indicates that yoghurt produced using strains capable of EPS synthesis exhibit better rheological properties, viscosity, and consistency than those made with strains that cannot synthesize these polymers.

The presence of proteins in yoghurts is very important because they improve viscosity, bind water, act as stabilizers, and limit syneresis during storage at low temperatures. In the dairy industry proteins in the form of powdered milk or whey proteins are added to modify the product’s consistency. Thus, the addition of proteins to yoghurt is beneficial for technological purposes. The texture of yoghurt is also positively influenced by the addition of polysaccharides [68] and inulin [24].

In the present study, due to legal limits [19] on the addition of lactoferrin (as well as its high cost), the level of added lactoferrin could not have been higher, as the main purpose of the study was to assess a product intended for the market. Therefore, the addition of lactoferrin caused no significant changes in texture parameters (Table 4). The texture parameters of fresh natural yoghurts supplemented with LF were as follows: firmness—3.35 N, consistency—2.01 mJ, cohesive strength—1.20 N, and dynamic viscosity—850 mPa·s. The values for the control yoghurts were similar (*p* > 0.05, Table 4).

The parameters were significantly influenced by the storage period. There was an increase in firmness (*p* ≤ 0.01), consistency (*p* ≤ 0.01) and dynamic viscosity (*p* ≤ 0.01), and a decrease in cohesive strength for both the yoghurt with added LF and the control yoghurt (Table 4).

Many authors report changes in the texture parameters of natural yoghurt. Similar tendencies during storage of natural yoghurt were observed by Moschopoulou et al. [69] and Domagała et al. [70], while Pawlos [71] and Bierzuńska et al. [72] reported slightly different relationships, with higher values obtained for the texture parameters. According to Das and Seth [73], the rheological characteristics of yoghurt enriched with colostrum may have been improved by the increased content of whey proteins in colostrum, as protein content is directly associated with the forces involved in the internal bonds of the product. The texture parameters of yoghurt increase with the share of dry matter, including protein, but also fat [74]. They are also associated with the capacity of the inoculant to produce slime [67]. The tendency of the viscosity of the yoghurts to increase with storage time in the present study is confirmed by Karam-Allah et al. [22], who evaluated stirred yoghurts fortified with lactoferrin colostrum and found lower viscosity in the control and experimental yoghurts than in the present study (by about 30%). The addition of lysozyme and lactoferrin to donkey milk also had no significant effect on the texture of yoghurt produced by Akal et al. [23]. Compared to the control yoghurt, the addition of the proteins caused a non-significant increase in firmness and the index of viscosity and a decrease in consistency and cohesiveness. Storage for 30 days increased most of the parameters of donkey yoghurt treated with LF, i.e., firmness by 4%, consistency by 25% and cohesiveness by 12%, while the index of viscosity decreased by 18%. Many authors [21,75] confirm an increase in firmness during storage, which results from shrinkage of the protein gel due to changes in pH. Therefore, the firmness of yoghurts depends mainly on the activity of lactic acid bacteria.

### 3.6. Water Activity

Water activity (a_w_) is the availability of the water contained in a product for microbes. The water activity parameter can be used to determine the course of biochemical reactions, the stability of the organoleptic characteristics of food, the development of microorganisms, and above all the storage stability of food products [45]. Olkowski et al. [76] reported that unfavourable reactions affecting food quality are more dependent on the state of the water than on its content in the product. In the present study, water activity generally ranged from 0.937 to 0.978 (Table 5). It was lower in the control yoghurt than in the fortified product: 0.937 vs. 0.948. The values increased during storage, which was confirmed statistically (*p* ≤ 0.05). The highest water activity was recorded for the yoghurt fortified with LF on the last day of storage (0.978). Water activity is rarely analysed in studies on yoghurt. Similar values for natural yoghurts have been reported by Cais-Sokolińska and Walkowiak-Tomczak [40], who obtained water activity at a level of 0.9776. Similar tendencies were noted in another study that took into account the storage period of products [45]. In general it can be stated that the higher the aw index, the faster microorganisms can multiply, using the water for their own processes. In the case of dairy products, one means of controlling water activity is to regulate their pH [77].

### 3.7. Water–Holding Capacity and Syneresis

Separation of whey from yoghurt is a natural phenomenon, although it is negatively perceived by consumers, who generally associate it with adverse changes in quality and as a sign of deterioration [45].

The water–holding capacity (WHC) of yoghurts is an indicator of their ability to retain serum (whey) in the gel structure [78]. The fresh control yoghurt and yoghurt with added LF had comparable WHC values, amounting to 86.25% and 87.00%, respectively (Table 5). However, a significant (*p* ≤ 0.05) influence of storage time was observed, with WHC deteriorating over storage time. On the last day of storage it was 10% lower than on day 0. This should also be linked to changes taking place in the texture of the yoghurt, e.g., firmness. In the case of WHC, similar tendencies were reported by Brodziak et al. [45], who analysed yoghurts enriched with WPC. As the amount of WPC increased, WHC decreased. It also decreased significantly during refrigerated storage, irrespective of the bacterial culture used. In a study by Bierzuńska et al. [72], the addition of polymerized whey protein (PWP) in the amount of 28% *w*/*v* significantly (*p* < 0.05) increased WHC (97.70%), while the addition of whey protein concentrate (WPC) in the amount of 5.62% *w*/*v* reduced it (92.41%, *p* < 0.05) in comparison with the control yoghurt (95.23%). Three weeks of refrigerated storage decreased the ability of WPC yoghurt to retain water by about 20%, while PWP yoghurt showed little difference (3%). The direction of the changes in the case of the use of WPC is also confirmed by Akalin et al. [79], and Kozioł et al. [80]. A study by Cais-Sokolińska and Walkowiak-Tomczak [40] also confirmed that the storage time of natural yoghurt significantly affects its WHC, obtaining values of over 90%. Ning et al. [81] reported 96% WHC in fresh natural yoghurt. Yoghurts analysed by Akal et al. [23] had WHC values at about half the level obtained in the present study. Yoghurt made from donkey milk, with and without the addition of proteins (lysozyme, lactoferrin and natamycin), had a very similar capacity, ranging from 38.82% to 39.25%. This parameter was not significantly affected by 30-day storage, although the WHC values increased (to 43%).

The present study also determined spontaneous whey syneresis, also called whey separation. This is an important phenomenon taking place during storage and is visible with the naked eye. According to Dimitrellou et al. [82], this defect can affect acceptance of the final product, due to an unfavourable appearance, and limit shelf life. It is caused in yoghurt by the loss of gel capacity to entrap the serum phase due to weakening of the gel network, resulting in separation of the whey [53]. It can be determined by the type of milk, the type of bacterial cultures, the acidity of the yoghurt, total solids, protein content, or hydrocolloid content. The presence of EPS also affects spontaneous whey separation, increasing it during storage [78,83]. This is a common, natural phenomenon in yoghurt, but in order to satisfy consumers, producers try to reduce it using various additives—polysaccharides (e.g., carrageenan, guar gum, xanthan gum, locust bean gum, or mixtures thereof), milk proteins in the form of skimmed milk powder, whey powder, whey protein concentrate (WPC), prebiotics (e.g., inulin), or dietary fibre [84]. In the present study, a comparable level of syneresis was observed in both types of yoghurt (Table 5). The addition of protein was too small to significantly affect syneresis. However, SWS changed significantly (*p* ≤ 0.01) during storage. In the control yoghurt it was 12× higher on day 28 of storage than on day 0 (1.2 vs. 0.1%), while in the experimental yoghurt it was 10× higher (1.0 vs. 0.1%). Comparable SWS values were obtained by Bierzuńska et al. [72] and by Cais-Sokolińska and Walkowiak-Tomczak [40] for natural yoghurt, with a higher WHC. The degree of syneresis increased significantly (*p* ≤ 0.05) over storage time in the control yoghurt (from 0.1% on day 0 to 1.5% on day 21) and in yoghurt with whey protein concentrate—WPC (from 0.1% on day 0 to 3.9% on day 21). The addition of polymerized whey protein (PWP) reduced syneresis to the level of the control yoghurt on day 0—the level of 0.0% was maintained over a 21-day period [72]. The degree of syneresis in the study by Cais-Sokolińska and Walkowiak-Tomczak [40] was 0.1% in the fresh control yoghurt made using *S. thermophilus*, *L. acidophilus*, and *Bifidobacterium animalis* ssp. *lactis*. Syneresis was not observed also after three weeks of storage. Increasingly proposed and used plant additives also affect the water–holding capacity and syneresis. According to Khalil et al. [85], the syneresis and WHC values of probiotic (*Bifidobacterium longum*) yoghurt flavoured with white sapote fruit (*Casimiroa edulis*) in the form of pulp were influenced by supplementation whether in fresh or stored samples.

### 3.8. Colour Parameters

The CIELab system (L*a*b*) is currently the most popular means of describing the colour of various food products and the basis for modern colour management systems. The L* (lightness), a* (change in the green-to-red range) and b* (change in the blue-to-yellow range) parameters were measured instrumentally. The yoghurt without added LF was lighter (97.75) than those with it (91.06, *p* > 0.05), probably due to the natural pink colour of LF (Table 6). The lightness of the yoghurts decreased over time, which was confirmed statistically (*p* ≤ 0.01), and was lowest on the final day of the study, i.e., day 28. The colour of fresh yoghurt with added LF took on values closer to red (parameter a*; *p* ≤ 0.01) and yellow (b*; *p* ≤ 005) compared to the control yoghurt. During storage time the yoghurts became both redder (change in a* towards 0, i.e., from −4.43 to −3.16 in the control yoghurt and from −2.04 to −0.85—in the fortified yoghurt) and yellower (b*, i.e., from 18.12 to 18.57 in the control yoghurt and from 17.68 to 18.12 in the fortified yoghurt). Previously published studies regarding LF in dairy products have not included instrumental measurement of colour, so it is not possible to compare the results directly with the findings of other authors. Pires et al. [86], in a study using natural yoghurt purchased in a shop, reported an L* value of 93, a* −3.5, and b* 9.8, with 3.3% fat content and pH 4.3. In the case of the L* and a* parameters, these values were similar to the results obtained in the present study for the fortified yoghurt. They also correspond with results obtained by Cais-Sokolińska and Walkowiak-Tomczak [40] for natural yoghurts produced by the authors (L* 89.7, a* −2.1). During three-week storage, however, the L* and a* values proved to be unstable, and that of L* decreased significantly (*p* ≤ 0.05). In another study, Cais-Sokolińska and Pikul [87] reported that as the storage time of yoghurts increased, the L*, a* and b* colour parameters decreased, which was confirmed in the present study only for lightness (L*). Changes in these parameters are also determined by factors other than additives, such as the course of pasteurization or changes in pH during storage. Bierzuńska et al. [72] reported that control yoghurts and yoghurts with added polymerized whey protein (PWP) became whiter with storage time, while those enriched with whey protein concentrate (WPC) became less white. Therefore it is difficult to draw clear conclusions regarding the direction of changes. Nevertheless, instrumental colour assessment can unquestionably be used to choose and optimize the conditions for the technological process. It should be noted, however, that instrumental colour assessment will not reflect consumers’ visual evaluation and acceptance of colour. Minor changes registered by the device will not be registered at all by the human eye.

### 3.9. Organoleptic Assessment

**Figure 4:**
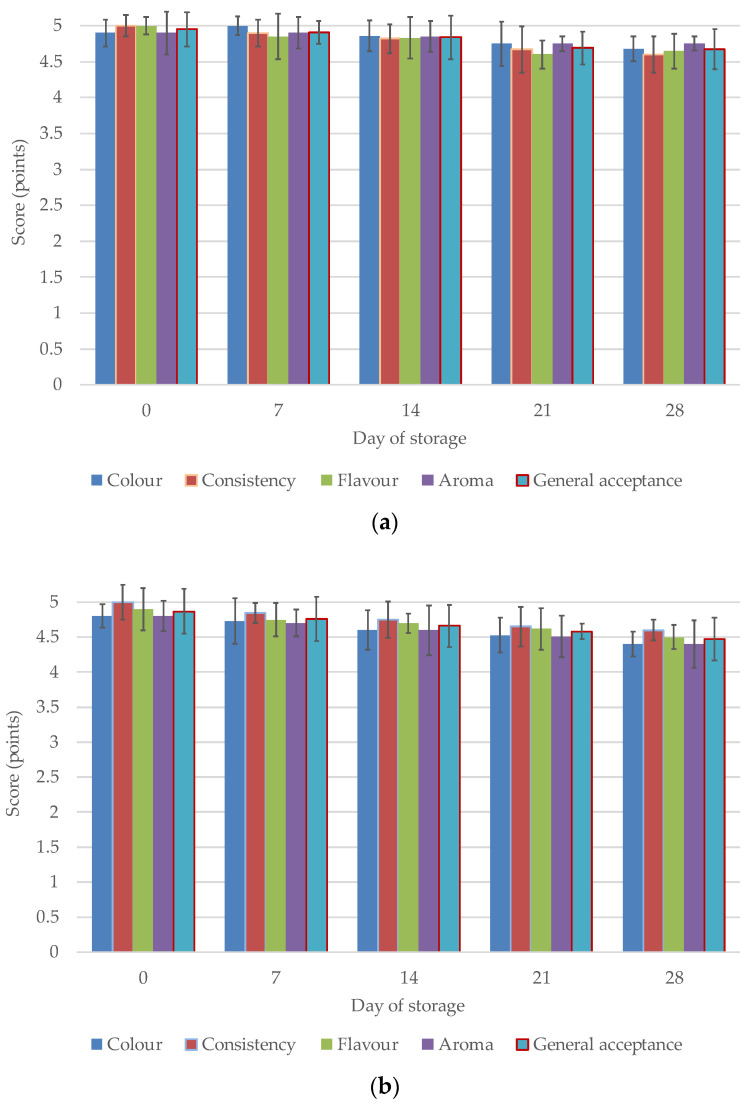
(**a**) Changes in organoleptic quality (colour, consistency, flavour, aroma and general acceptance) of the plain, natural yoghurts during 28 days of storage in the refrigerated conditions. (**b**) Changes in organoleptic quality (colour, consistency, flavour, aroma and general acceptance) of the yoghurts fortified in lactoferrin during 28-days of storage in the refrigerated conditions.

A very important element of the evaluation of a new product is consumer surveys [22,55,88]. The organoleptic assessment of the yoghurts took into account their colour, flavour, consistency, aroma and general acceptance. These characteristics directly depend on the acidity of the yoghurt, including lactic acid content, and its content of free fatty acids and volatile compounds. Based on organoleptic evaluation, the consumer is able to assess the quality of the purchased product, but in the case of yoghurt, this is only possible after the package has been opened. The yoghurts fortified with lactoferrin received high scores, comparable to the scores for the control yoghurt (Figure 4a,b). The fresh experimental yoghurt received the same total score as the control yoghurt, i.e., 4.9 points/5.0 max. The effect of the additive therefore did not prove to be significant, and the comparable, high results obtained can be considered satisfactory. It is important for a new product to have high consumer acceptance. In general, the scores assigned for organoleptic traits decreased during the storage period, reaching an average of 4.68 points/5.00 max. However, the effect proved to be statistically non-significant (*p* > 0.05), i.e., there were no significant, negative changes in perception. The tendencies found for natural yoghurt are confirmed in a study by Arslaner et al. [56]. Cais-Sokolińska and Walkowiak-Tomczak [40] reported full acceptance of natural yoghurt immediately after production, but after three weeks of storage the acceptance level was 14%. ‘Dislike’ responses were given by two of thirteen panellists. According to Karam-Allah et al. [22], stirred yoghurt fortified with lactoferrin colostrum had statistically (*p* ≤ 0.05) higher scores for body, texture, and appearance than the control sample, but lower scores for flavour. Thus, the addition of lactoferrin improved the organoleptic traits of the product. This is consistent with results reported by Das and Seth [73], who reported that curd samples fortified with colostrum whey powder had higher scores than the control yoghurt in terms of body and texture, colour, appearance, and overall acceptability. Studies performed using different dairy products also found that the addition of lactoferrin [23], lysozyme [89], and natamycin [90] had no adverse effects on the organoleptic properties of products. Akalin et al. [79] reported no significant differences between yoghurts with different amounts of WPC in terms of appearance, aroma, flavour, and overall acceptability during storage (*p* > 0.05). Zakaria et al. [57] conducted only a seven-day evaluation of yoghurt fortified with lactoferrin, which on the first day after production received lower scores than the control yoghurt. On the seventh day of storage, LF-fortified yoghurt received lower scores than during analysis the day after production, but none of these tendencies was confirmed statistically.

## 4. Conclusions

Enrichment of yoghurt with lactoferrin did not cause significant changes in most of the physicochemical properties analysed (except for total protein, content of dry matter and lactoferrin, and colour parameters) or in its microbiological or organoleptic properties. In the case of content of basic nutrients (total protein and dry matter), the bioactive additive itself (LF content), texture parameters (firmness, consistency, and dynamic viscosity), and physical characteristics (acidity, water activity, water-holding capacity, spontaneous whey syneresis and colour parameters), a significant influence of storage time was observed. The results are satisfactory, and indicate high acceptance and high stability of the yoghurts fortified in LF.

Lactoferrin, as an integral component of milk, is safe, and, moreover, it has proven multi-faceted health-promoting effects on the human body. The proposed product—yoghurt fortified with LF—may be of interest to health-conscious consumers. An improvement of biological value increases interest in food products. Analysis of previously published studies on whey proteins suggests that further studies should aim to determine the population groups that could derive the greatest health benefits from consuming lactoferrin-fortified yoghurts. Biochemical studies, which usually assess the current state of the body, and genetic studies, which can serve as a prognostic indicator, could be used to identify the target audience for lactoferrin-fortified yoghurts and fully exploit their preventive properties. Next steps for further research could include investigating the effects of different forms of lactoferrin on yoghurt, such as holo and apo, and exploring the potential benefits of encapsulation to improve bioavailability and extend the presence of bioactive substances in the body. Additionally, further research could examine the impact of LF on other dairy products, and investigate the co-effects of other bioactive substances on the physicochemical and organoleptic properties of dairy products. Ultimately, research in this area could help to develop the innovative and appealing dairy products that meet the demands of health-conscious consumers.

## Figures and Tables

**Table 1:** Results of basic chemical composition analysis of yoghurts during 28 days of storage in the refrigerated conditions (mean ± SD).

Yoghurt Type	Day of Storage	Number of Samples *	Energy (kJ/100 g)	Total Protein (%)	Fat (%)	Dry Matter (%)
Plain, natural yoghurt	0	3	63 ± 3	3.14 ^bx^ ± 0.05	3.26 ± 0.06	12.27 ^Bx^ ± 0.26
	7	3	63 ± 4	3.12 ^bx^ ± 0.08	3.22 ± 0.08	12.18 ^Bx^ ± 0.20
	14	3	62 ± 3	3.07 ^abx^ ± 0.11	3.16 ± 0.12	11.99 ^Bx^ ± 0.31
	21	3	63 ± 4	2.98 ^ax^ ± 0.09	3.12 ± 0.10	11.34 ^Ax^ ± 0.25
	28	3	62 ± 5	2.88 ^ax^ ± 0.12	3.10 ± 0.15	10.95 ^Ax^ ± 0.43
Yoghurt with lactoferrin	0	3	64 ± 2	3.33 ^by^ ± 0.04	3.25 ± 0.07	12.36 ^Cy^ ± 0.30
	7	3	64 ± 3	3.22 ^by^ ± 0.07	3.23 ± 0.11	12.29 ^BCy^ ± 0.37
	14	3	64 ± 4	3.16 ^by^ ± 0.13	3.18 ± 0.14	12.12 ^By^ ± 0.40
	21	3	63 ± 4	3.08 ^ay^ ± 0.11	3.15 ± 0.12	11.49 ^Ay^ ± 0.33
	28	3	63 ± 6	3.00 ^ay^ ± 0.10	3.11 ± 0.16	11.10 ^Ay^ ± 0.52

*—new samples were taken in the following days of storage. The total number of samples covered by the research in this field is the sum of all those listed. ^a, b, A, B, C^—differences between the basic chemical composition within a yoghurt type; ^a, b^—significant differences at *p* ≤ 0.05; ^A, B, C^—significant differences at *p* ≤ 0.01. ^x, y^—differences between the basic chemical composition between a yoghurt type within a day of storage; ^x, y^—significant differences at *p* ≤ 0.05.

**Table 2:** Lactoferrin content in yoghurts during 28 days of storage in the refrigerated conditions (mean ± SD).

Yoghurt Type	Number of Samples *	Day of Storage	Lactoferrin (mg/100 g)
Plain, natural yoghurt	3	0	47.69 ^bX^ ± 2.95
	3	7	47.12 ^bX^ ± 2.51
	3	14	45.24 ^abX^ ± 4.25
	3	21	44.01 ^abX^ ± 4.90
	3	28	41.17 ^aX^ ± 4.86
Yoghurt with lactoferrin	3	0	131.15 ^bY^ ± 9.17
	3	7	130.37 ^bY^ ± 12.01
	3	14	129.20 ^bY^ ± 11.38
	3	21	126.64 ^abY^ ± 14.10
	3	28	123.55 ^aY^ ± 14.84

*—new samples were taken in the following days of storage. The total number of samples covered by the research in this field is the sum of all those listed. ^a, b^—differences between a day of storage within a yoghurt type; ^a, b^—significant differences at *p* ≤ 0.05. ^X, Y^—differences between a yoghurt type within a day of storage; ^X, Y^—significant differences at *p* ≤ 0.01.

**Table 3:** Microbiological evaluation of the analysed yoghurts during 28 days of storage in the refrigerated conditions (cfu/g, mean).

Yoghurt Type	Day of Storage	Number of Samples *	Total Bacteria Count	Total Number of Fungi	Total Number of Lactic Acid Bacteria	Total Number of Bacteria of the Genus *Clostridium* sp.	Total Number of *Coli* Bacteria (Endo)	Total Number of Faecal Coliforms (mFC)	Presence of *Salmonella* Bacilli	Presence of *Campylobacter* Bacilli
Plain, natural yoghurt	0	3	0	0	6.9 × 10^7^	0	0	0	0	0
	7	3	0	0	7.6 × 10^7^	0	0	0	0	0
	14	3	<1.5 × 10^1 Y^	0	6.4 × 10^7^	0	0	0	0	0
	21	3	<1.5 × 10^1 Y^	0	5.9 × 10^7^	0	0	0	0	0
	28	3	<1.5 × 10^1 Y^	0	5.2 × 10^7^	0	0	0	0	0
Yoghurt with lactoferrin	0	3	0	0	7.1 × 10^7^	0	0	0	0	0
	7	3	0	0	7.4 × 10^7^	0	0	0	0	0
	14	3	0 ^X^	0	6.5 × 10^7^	0	0	0	0	0
	21	3	0 ^X^	0	5.8 × 10^7^	0	0	0	0	0
	28	3	0 ^X^	0	5.0 × 10^7^	0	0	0	0	0

*—new samples were taken in the following days of storage. The total number of samples covered by the research in this field is the sum of all those listed. “0”—means “not detected”. ^X, Y^—differences between a yoghurt type within a day of storage; ^X, Y^—significant differences at *p* ≤ 0.01.

**Table 4:** Results of texture analysis of yoghurts during 28 days of storage in the refrigerated conditions (mean ± SD).

Yoghurt Type	Day of Storage	Number of Samples *	Firmness (N)	Consistency (mJ)	Cohesive Strength (N)	Dynamic Viscosity (mPa·s)
Plain, natural yoghurt	0	3	3.89 ^A^ ± 2.17	2.38 ^A^ ± 0.40	1.18 ± 0.12	812 ± 86
	7	3	4.43 ^B^ ± 1.51	2.82 ^A^ ± 0.14	1.16 ± 0.10	950 ± 102
	14	3	4.73 ^AB^ ± 1.34	3.09 ^B^ ± 0.23	1.13 ± 0.10	1130 ± 76
	21	3	6.23 ^C^ ± 1.02	3.85 ^C^ ± 0.29	1.10 ± 0.14	1292 ± 100
	28	3	6.45 ^C^ ± 1.10	3.73 ^C^ ± 0.42	1.07 ± 0.15	1975 ± 143
Yoghurt with lactoferrin	0	3	3.35 ^A^ ± 2.09	2.01 ^B^ ± 0.28	1.20 ± 0.09	850 ± 62
	7	3	4.21 ^B^ ± 1.38	2.47 ^A^ ± 0.60	1.17 ± 0.13	910 ± 57
	14	3	4.08 ^AB^ ± 1.21	2.26 ^B^ ± 0.35	1.13 ± 0.12	1090 ± 91
	21	3	6.09 ^C^ ± 1.42	4.06 ^C^ ± 0.55	1.13 ± 0.11	1209 ± 126
	28	3	6.27 ^C^ ± 1.35	4.08 ^C^ ± 0.34	1.10 ± 0.16	1370 ± 107

*—new samples were taken in the following days of storage. The total number of samples covered by the research in this field is the sum of all those listed. ^A, B, C^—differences between the texture parameters within a yoghurt type; ^A, B, C^—significant differences at *p* ≤ 0.01.

**Table 5:** Water activity, water-holding capacity and syneresis in yoghurts during 28 days of storage in the refrigerated conditions (mean ± SD).

Yoghurt Type	Day of Storage	Number of Samples *	Water Activity	Water-Holding Capacity—WHC (%)	Spontaneous Whey Syneresis—SWS (%)
Plain, natural yoghurt	0	3	0.937 ^a^ ± 0.008	86.25 ^c^ ± 0.62	0.1 ^A^ ± 0.0
	7	3	0.945 ^a^ ± 0.007	82.94 ^b^ ± 0.53	0.5 ^B^ ± 0.1
	14	3	0.960 ^ab^ ± 0.013	80.79 ^a^ ± 0.94	0.8 ^B^ ± 0.2
	21	3	0.964 ^b^ ± 0.009	80.05 ^a^ ± 1.68	1.0 ^BC^ ± 0.3
	28	3	0.971 ^b^ ± 0.005	78.04 ^a^ ± 1.15	1.2 ^C^ ± 0.2
Yoghurt fortified in lactoferrin	0	3	0.948 ^a^ ± 0.006	87.00 ^c^ ± 0.34	0.1 ^A^ ± 0.1
	7	3	0.957 ^a^ ± 0.014	84.46 ^b^ ± 0.60	0.4 ^B^ ± 0.1
	14	3	0.952 ^a^ ± 0.009	81.63 ^a^ ± 0.87	0.5 ^B^ ± 0.3
	21	3	0.961 ^a^ ± 0.010	80.17 ^a^ ± 1.79	0.8 ^BC^ ± 0.2
	28	3	0.978 ^b^ ± 0.007	79.02 ^a^ ± 1.52	1.0 ^C^ ± 0.3

*—new samples were taken in the following days of storage. The total number of samples covered by the research in this field is the sum of all those listed. ^a, b, A, B, C^—differences between the texture parameters within a yoghurt type; ^a, b^—significant differences at *p* ≤ 0.05; ^A, B, C^—significant differences at *p* ≤ 0.01.

**Table 6:** Colour parameters of yoghurts during 28 days of storage in the refrigerated conditions (mean ± SD).

Yoghurt Type	Day of Storage	Number of Samples *	L*	a*	b*
Plain, natural yoghurt	0	3	97.75 ^By^ ± 1.69	−4.43 ^AX^± 0.18	18.12 ^ay^ ± 0.14
	7	3	96.48 ^By^ ± 1.37	−4.01 ^AX^ ± 0.23	18.15 ^ay^ ± 0.10
	14	3	96.01 ^By^ ± 1.25	−3.82 ^AX^ ± 0.27	18.32 ^ay^ ± 0.08
	21	3	94.62 ^ABy^ ± 1.90	−3.57 ^ABX^ ± 0.37	18.45 ^aby^ ± 0.11
	28	3	93.71 ^Ay^ ± 1.86	−3.16 ^BX^ ± 0.38	18.57 ^by^ ± 0.13
Yoghurt fortified in lactoferrin	0	3	91.06 ^Bx^ ± 1.71	−2.04 ^AY^ ± 0.22	17.68 ^ax^ ± 0.20
	7	3	90.43 ^Bx^ ± 1.15	−1.75 ^AY^ ± 0.16	17.73 ^ax^ ± 0.14
	14	3	90.02 ^Bx^ ± 1.38	−1.44 ^ABY^ ± 0.29	17.84 ^ax^ ± 0.15
	21	3	88.31 ^ABx^ ± 1.20	−1.18 ^BY^ ± 0.36	17.96 ^abx^ ± 0.20
	28	3	87.15 ^Ax^ ± 1.42	−0.85 ^BY^ ±0.45	18.12 ^bx^ ± 0.23

L*—lightness; a*—a change in the green to red range; b*—a change in the blue to yellow range (the CIELab system). *—new samples were taken in the following days of storage. The total number of samples covered by the research in this field is the sum of all those listed. ^a, b, A, B^—differences between a day of storage within a yoghurt type; ^a, b^—significant differences at *p* ≤ 0.05; ^A, B^—significant differences at *p* ≤ 0.01. ^x, y, X, Y^—differences between a yoghurt type within a day of storage; ^x, y^—significant differences at *p* ≤ 0.05; ^X, Y^—significant differences at *p* ≤ 0.01.

## Data Availability

Not applicable.
